# Data on lateral collection length of charge carriers depending on pre-white-light soaking process for metal mesh transparent electrode based Cu(In,Ga)Se_2_ solar cells

**DOI:** 10.1016/j.dib.2019.104407

**Published:** 2019-08-20

**Authors:** Sangyeob Lee, Jiseong Jang, Kyung Soo Cho, Yong-Jun Oh, Ki-Ha Hong, Choong-Heui Chung

**Affiliations:** a Department of Materials Science and Engineering, Hanbat National University, Daejeon, 34158, Republic of Korea; b Department of Materials and Manufacturing Engineering, Hanbat National University, Daejeon, 34158, Republic of Korea

**Keywords:** Lateral collection length, Solar cells, CIGS, Transparent electrodes, Metal mesh, Silver nanowire

## Abstract

The authors have recently reported silver nanowire based Cu(In,Ga)Se_2_ solar cells [1,2]. Metal mesh based transparent electrodes other than the silver nanowire can be also employed or have a potential to provide a better performance for the solar cells. To select a suitable electrode for a solar cell among metal meshes, it is required to have data on the lateral collection length of charge carriers in the targeted cell. The method to determine the lateral collection has been reported in our previous publication [3]. Here, we report data on the effect of the light intensity during pre-white-light soaking on the lateral charge collection length for metal mesh transparent electrode based Cu(In,Ga)Se_2_ solar cells.

**Table:** Specifications Table

Subject	Electrical engineering
Specific subject area	Solar cells
Type of data	Table and Figures
How data were acquired	Keithley 2401 source meter under light illumination
Data format	Raw, and Analyzed
Parameters for data collection	The light intensity of pre-white-light soaking process
Description of data collection	The photocurrent values were measured from a short-circuit conditioned device, with a structure of Al/Ni/CdS/Cu(In,Ga)Se_2_/Mo under various light illumination conditions. Al/Ni is disk-shaped, and CdS/Cu(In,Ga)Se_2_/Mo is planar stacked.
Data source location	Hanbat National University, Daejeon 34158, Republic of Korea
Data accessibility	The data are with this article
Related research article	Author's name: Sangyeob Lee et al. Title: Determination of the lateral collection length of charge carriers for silver-nanowire-electrode-based Cu(In,Ga)Se_2_ thin-film solar cells Journal: Solar Energy https://doi.org/10.1016/j.solener.2019.01.059

**Table:** 

**Value of the data** • The solar light intensity is not fixed but varied during day time. Therefore, it is required to have data regarding the effect of solar light intensity on the lateral collection length of charge carriers to properly design a metal mesh electrode for solar cells. • The data can be useful for device/process engineers who are responsible for determining a type of transparent electrode for a solar cell. Depending on the lateral collection length of chare carriers, it can be determined which one, mesh type or thin film type, is the most suitable for a targeted solar cell. • In metal mesh transparent electrode based Cu(In,Ga)Se_2_ (CIGS) solar cells, charge carriers are laterally collected along a buffer layer, CdS in this work. Alternative buffer layers without a toxic element have been studied elsewhere, and they will show different values of the lateral collection length from CdS. The lateral collection length will be one of criteria in selecting an alternative buffer layer for metal mesh transparent electrode based CIGS solar cells.

## 1 Data

**Fig. 1:**
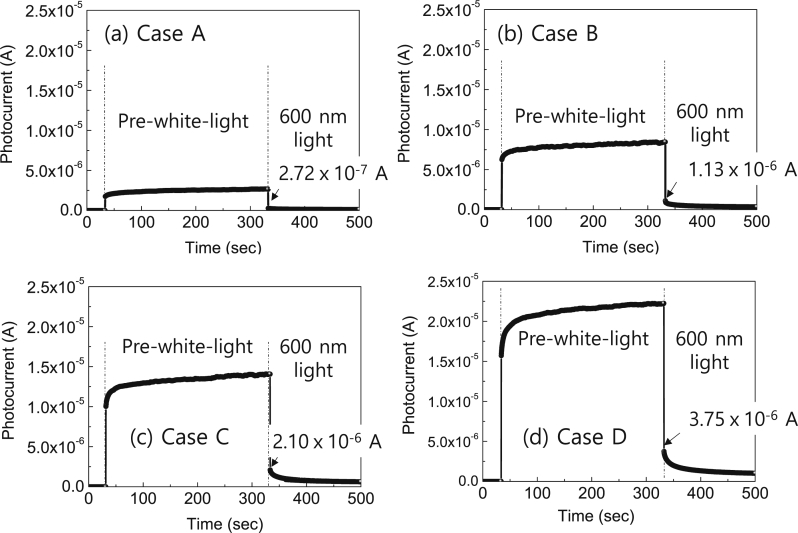
The variation of the measured photocurrents with four different illumination conditions of case A, B, C, and D.

**Fig. 2:**
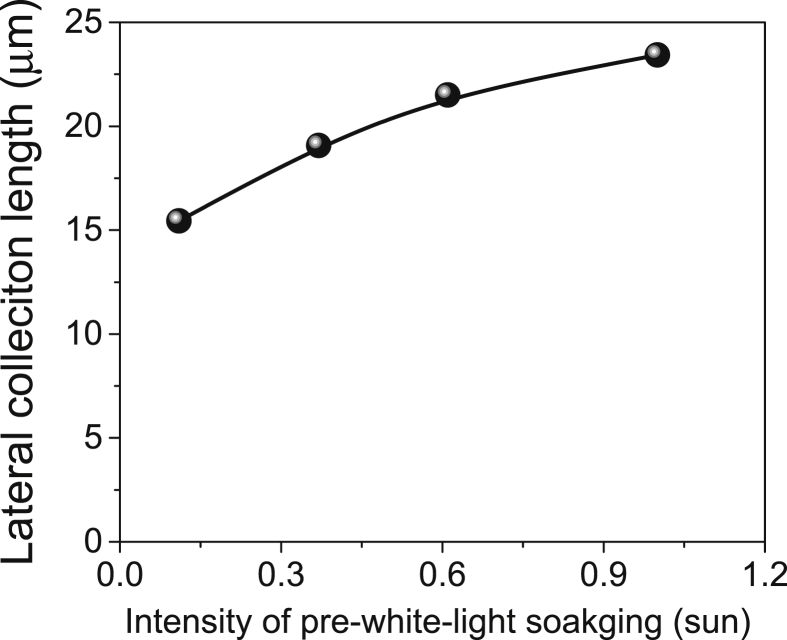
The determined lateral collection lengths as a function of the intensity of pre-white-light soaking.

Table 1 summarize the intensity of pre-white-light soaking and 600 nm-narrow-bandwidth-light, and the photocurrent upon illumination of 600 nm-narrow-bandwidth-light immediately after a corresponding the pre-white-light soaking for four cases A, B, C, and D. The diameter of a disk-shaped Al/Ni top electrode formed on CdS/CIGS/Mo planar stack was 1.75 mm. The reflectance of the light off the front CdS surface was 0.09. Fig. 1 shows the variation of the measured photocurrents with illumination conditions for four cases A, B, C, and D. With increasing the intensity of pre-white-light soaking, the photocurrent upon illumination of 600 nm-narrow-bandwidth-light immediately after a corresponding the pre-white-light soaking increased. Fig. 2 shows the determined lateral collection lengths as a function of the intensity of pre-white-light soaking. The value increased from 15.4 to 23.4 μm with increasing the intensity of pre-white-light soaking from 0.11 to 1.0 sun. The lateral collection length data will be useful to design a suitable network for silver nanowire based CIGS thin film solar cells [1], [2].

**Table 1:** The intensity of pre-white-light soaking and 600 nm-narrow-bandwidth-light, and the photocurrent upon illumination of 600 nm-narrow-bandwidth-light immediately after a corresponding the pre-white-light soaking for four cases A, B, C, and D.

Diameter = 175 mm of a disk shaped Al/Ni formed on a CdS/CIGS/Mo stack			
Case	Pre-white-light soaking	600 nm narrow bandwidth light	
	Intensity 1.0 sun = 100 mW/cm^2^	Intensity (W/cm^2^)	Photocurrent (A)
A	0.11 sun	7.3	2.72 × 10^−7^
B	0.37 sun	24.5	1.13 × 10^−6^
C	0.61 sun	40.4	2.10 × 10^−6^
D	1.0 sun	66.2	3.75 × 10^−6^

## 2 Experimental design, materials, and methods

The measured device has a structure of Al/Ni/CdS/CIGS/Mo. Al/Ni is disk-shaped, and formed by e-beam evaporation through a shadow mask on the CdS/CIGS/Mo planar stack. The reflectance of the light off the front CdS surface was measured using an UV–Vis spectrometer. Photocurrents were measured from the short-circuited device under light illumination generated by a solar simulator. The intensity of white-light, employed for pre-white-light soaking process, shining the device was controlled by adjusting the device-to-solar simulator distance. The 600 nm-narrow-bandwidth-light, which was employed to determine the lateral collection length of charge carriers, was generated by inserting a 600 nm-band pass filter between the device and the solar simulator. The lateral collection lengths were determined using equation (4) of our previous work [3].
